# Development and evaluation of novel salt‐tolerant *Eucalyptus* trees by molecular breeding using an *RNA‐Binding‐Protein* gene derived from common ice plant (*Mesembryanthemum crystallinum* L.)

**DOI:** 10.1111/pbi.13016

**Published:** 2018-10-12

**Authors:** Ngoc‐Ha Thi Tran, Taichi Oguchi, Nobuhumi Akatsuka, Etsuko Matsunaga, Akiyoshi Kawaoka, Akiyo Yamada, Yoshihiro Ozeki, Kazuo N. Watanabe, Akira Kikuchi

**Affiliations:** ^1^ Graduate School of Life and Environmental Sciences University of Tsukuba Tsukuba Ibaraki Japan; ^2^ Tsukuba Plant‐Innovation Research Center University of Tsukuba Tsukuba Ibaraki Japan; ^3^ Faculty of Life and Environmental Sciences University of Tsukuba Tsukuba Ibaraki Japan; ^4^ Department of Biotechnology Tokyo University of Agriculture and Technology Tokyo Japan; ^5^ Agri‐Biotechnology Research Laboratory Nippon Paper Industries Co., Ltd. Tokyo Japan

**Keywords:** RNA binding protein, common ice plant, *Mesembryanthemum crystallinum*, salt tolerance, *Eucalyptus camaldulensis*

## Abstract

The breeding of plantation forestry trees for the possible afforestation of marginal land would be one approach to addressing global warming issues. Here, we developed novel transgenic *Eucalyptus* trees (*Eucalyptus camaldulensis* Dehnh.) harbouring an *RNA‐Binding‐Protein* (*McRBP*) gene derived from a halophyte plant, common ice plant (*Mesembryanthemum crystallinum* L.). We conducted screened‐house trials of the transgenic *Eucalyptus* using two different stringency salinity stress conditions to evaluate the plants’ acute and chronic salt stress tolerances. Treatment with 400 mM NaCl, as the high‐stringency salinity stress, resulted in soil electrical conductivity (EC) levels >20 mS/cm within 4 weeks. With the 400 mM NaCl treatment, >70% of the transgenic plants were intact, whereas >40% of the non‐transgenic plants were withered. Treatment with 70 mM NaCl, as the moderate‐stringency salinity stress, resulted in soil EC levels of approx. 9 mS/cm after 2 months, and these salinity levels were maintained for the next 4 months. All plants regardless of transgenic or non‐transgenic status survived the 70 mM NaCl treatment, but after 6‐month treatment the transgenic plants showed significantly higher growth and quantum yield of photosynthesis levels compared to the non‐transgenic plants. In addition, the salt accumulation in the leaves of the transgenic plants was 30% lower than that of non‐transgenic plants after 15‐week moderate salt stress treatment. There results suggest that *McRBP* expression in the transgenic *Eucalyptus* enhances their salt tolerance both acutely and chronically.

## Introduction

Global warming is a common problem for all humankind, and the rapid increase of carbon dioxide emissions to the atmosphere since the Industrial Revolution is considered to be one of its main causes (UNFCCC, 1992). Forests have been playing an important role in the global carbon cycle as part of the carbon assimilation on land, but this area has gradually decreased by approx. 3.3 million hectares each year from 2010 to 2015 (FAO, 2015). Under this situation, forest plantations have played an important role in the efforts to compensate for the loss of natural forest with the sharing 7% of global forest area (Brockerhoff *et al*., 2013; FAO, 2015; Keenan *et al*., 2015).

*Eucalyptus* trees are currently the most important forestry plantation trees. The genus *Eucalyptus* (Myrtaceae) consists of least 600 species of flowering trees and shrubs. Most species of *Eucalyptus* are native to Australia, but additional species are found in Papua New Guinea, Indonesia, and on the island of Mindanao in the Philippines (Nishimura, 1987). As plantation forestry trees, about 10 species of fast‐growing *Eucalyptus* trees have been used for plantations throughout the tropics and subtropics in more than 90 countries across South America, Europe, Africa, China, the Indian subcontinent, and elsewhere (Booth, 2013; Eldridge *et al*., 1994; Nishimura, 1987). The global area of the *Eucalyptus* plantation reached approx. 19.6 million hectares in 2008 (Trabado, 2008), and was estimated to account for approx. 7% of the total industrial plantation forest area. One economically important species of *Eucalyptus*,* E. camaldulensis* Dehnh., has been widely cultivated for pulpwood, firewood, timber, shelterbelt, and essential oil (Boland *et al*., 2006; CAB‐International, 2000; Doran and Brophy, 1990; Nishimura, 1987) since the nineteenth century and is still an important plantation tree today. Methods for the Agrobacterium‐mediated transformation of several species of *Eucalyptus*, including *E. globulus* and *E. camaldulensis* Dehnh., have been reported (Chen *et al*., 2006; Matsunaga *et al*., 2012; Mullins *et al*., 1997; Prakash and Gurumurthi, 2009; Spokevicius *et al*., 2005; Tournier *et al*., 2003).

On another front, global climate change has made the area of salinity lands broader. The global salinity land area was approx. 932.2 million hectares (Rengasamy, 2006), or approx. 20% of the total cultivated land and 33% of the irrigated agricultural lands, (Munns, 2005; Shrivastava and Kumar, 2015). Besides, the global salinity land area was increasing by approx. 10% each year, and it was estimated that at this rate is more than 50% of the land available for agriculture will be lost by the year 2050 (Jamil *et al*., 2011).

We have speculated that the breeding of abiotic stress‐tolerant plantation trees could be one way to combat global climate change and contribute to the sustainable development of humankind. We have conducted molecular breeding of abiotic stress‐tolerant *Eucalyptus* trees using genetic resources of abiotic stress‐tolerant genes from halobacteria and/or halophytes (Kikuchi *et al*., 2009; Matsunaga *et al*., 2012; Oguchi *et al*., 2014; Tran *et al*., 2018; Yu *et al*., 2009, 2013a,b,c,d). *Mangrin* was found to be a candidate salt‐tolerant gene isolated from a cDNA library from suspension‐cultured cells of a mangrove plant, *Bruguiera sexangula* by a functional screening for cDNA encoding proteins essential for salt tolerance (Yamada *et al*., 2002). It was reported that the ectopic expression of *Mangrin* in bacteria, yeast, and tobacco suspension‐cultured cells improved these organisms’ salt tolerance (Yamada *et al*., 2002). We have also reported the development and evaluation of transgenic *E. camaldulensis* harbouring *Mangrin*, and we evaluated the plants’ salt tolerance in contained culture room cultivations and semi‐confined screen‐house trials (Lelmen *et al*., 2010; Yu *et al*., 2013d).

Here we report our development and evaluation of a novel salt‐tolerant *E. camaldulensis* harbouring *McRBP* (an RNA‐binding protein) derived from the common ice plant (*Mesembryanthemum crystallinum* L.). The common ice plant is a halophyte native to north and south‐western Africa, and it can grow well under severe salinity conditions (Adams *et al*., 1998; Bohnert and Cushman, 2000; CAB‐International, 2018; Cosentino *et al*., 2010; Kloot, 1983; Oh *et al*., 2015). *McRBP* was isolated by the same functional screening as used for the screening of *Mangrin*, and coded a protein of 306 amino acid residues, including two eukaryote RNA‐recognition motifs (RRM_1; Pfam identifier PF00076). RRM_1 motifs are suspected to be essential motifs for the improvement of salt tolerance in *Escherichia coli*, because *E. coli* expressing the partial protein of residues 127–306 of McRBP including two tandem RRM_1 motifs showed almost equivalent growth property to that expressing the full length McRBP in medium containing 650 mM NaCl (Yamada *et al*., 2011). The RRM_1 motif is widely preserved from bacteria to eukaryotes and is known to be related to the stability of RNA under low temperature conditions, but there has been no report on its relation to salt tolerance (Birney *et al*., 1993; Kupsch *et al*., 2012; Tillich *et al*., 2009). On the other hand, it has been reported that bacterial cold shock proteins (CSPs) exhibiting RNA chaperone activity improved not only low temperature tolerance but also other abiotic stresses such as drought in plants (Castiglioni *et al*., 2008). Our preliminary results suggested that young plantlets of the transgenic *E. camaldulensis* showed higher salt tolerance compared to non‐transgenic control plants under a contained culture room condition (NEDO, 2013). In this study, we aimed to evaluate the RNA chaperone activity in McRBP and its relation to salt tolerance in young trees of transgenic *E. camaldulensis* growing in a semi‐confined screen house.

## Results

### McRBP behaves as an RNA chaperone

**Figure 1:**
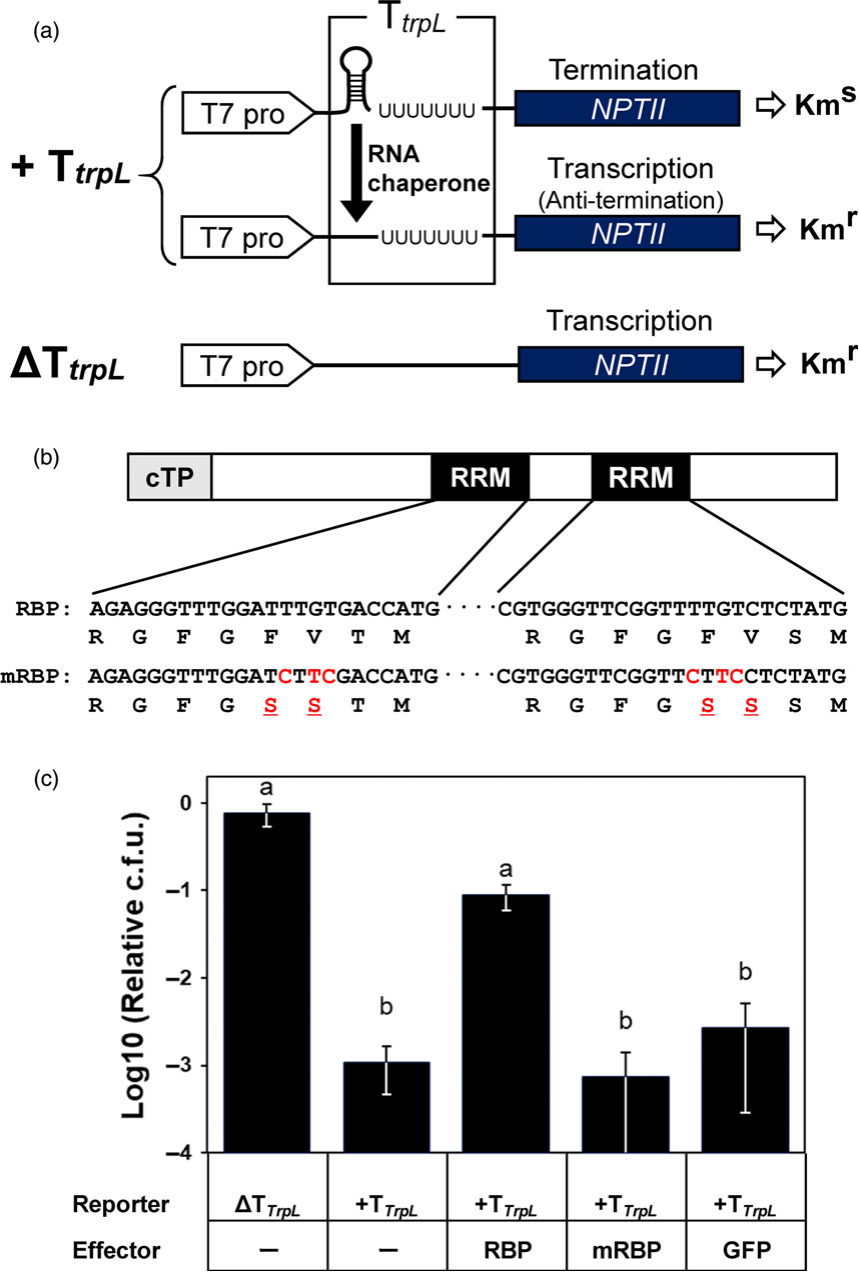
Transcription anti‐termination *in vivo* assay. (a) Schematic diagram of *NPTII* termination cassettes by a ρ‐independent *trpL* terminator. ∆T*trpL*, non‐ρ‐independent *trpL* terminator; Km^R^, kanamycin‐resistant; Km^S^, kanamycin‐sensitive; *NPTII*,* neomycin transferase II*; T7 pro, T7 promoter; T*trpL*, ρ‐independent *trpL* terminator. (b) Schematic diagram of the point mutations in RRM motifs of McRBP. cTP, chloroplast transit peptide; mRBP, mutant of RBP; RBP, RNA‐Binding‐Protein; RRM, RNA‐recognition motif. (c) Effect of McRBP on the kanamycin resistance of transgenic *Escherichia coli* carrying different groups of plasmids. The different letters at the top of the bars indicate significant differences among clones by the Tukey‐HSD test (α = 0.05). Error bars in (c) standard error.

*McRBP* was a novel salt‐tolerant gene that was isolated by the functional screening for cDNAs encoding proteins essential for salt tolerance using *E. coli* (Yamada *et al*., 2002, 2011). It was reported that *McRBP* overexpression enhanced the salt tolerance not only in *E. coli* but also yeast, e.g., the transgenic yeast over‐expressing *McRBP* showed clearly higher growth property than that of not expressing *McRBP* in the culture medium containing 1 m NaCl (Yamada *et al*., 2011). A search of the Pfam database predicted that McRBP contained two RNA recognition motifs designated RRM_1 (PF00076). In addition, the conserved motif alignment analysis by the SALAD database revealed that AtCP31A (TAIR ID: AT4G24770) and AtCP31B (AT5G50250) were the proteins most conserved with McRBP in *Arabidopsis thaliana* (Mihara *et al*., 2010) (Figure S1a). It was reported that AtCP31A and AtCP31B functioned in the stability of chloroplast RNA via the two conserved RRM_1 motifs (Tillich *et al*., 2009). Next, therefore, we evaluated the RNA chaperone activity of the McRBP. The RNA chaperone activity was assessed by means of a transcription anti‐termination assay (Phadtare *et al*., 2003) (Figure 1). The results of the transcription anti‐termination assay indicated that McRBP significantly suppressed the termination activity of the *trpL* terminator, while no anti‐termination activities were observed in the McRBP with mutations in amino acid residuals conserved in the RRM_1 motifs (Figures 1b,c, and S1b). These results suggested that McRBP behaves as an RNA chaperone *in vivo*.

### Copy number and expression level of *McRBP* gene in transgenic *Eucalyptus camaldulensis*

**Figure 2:**
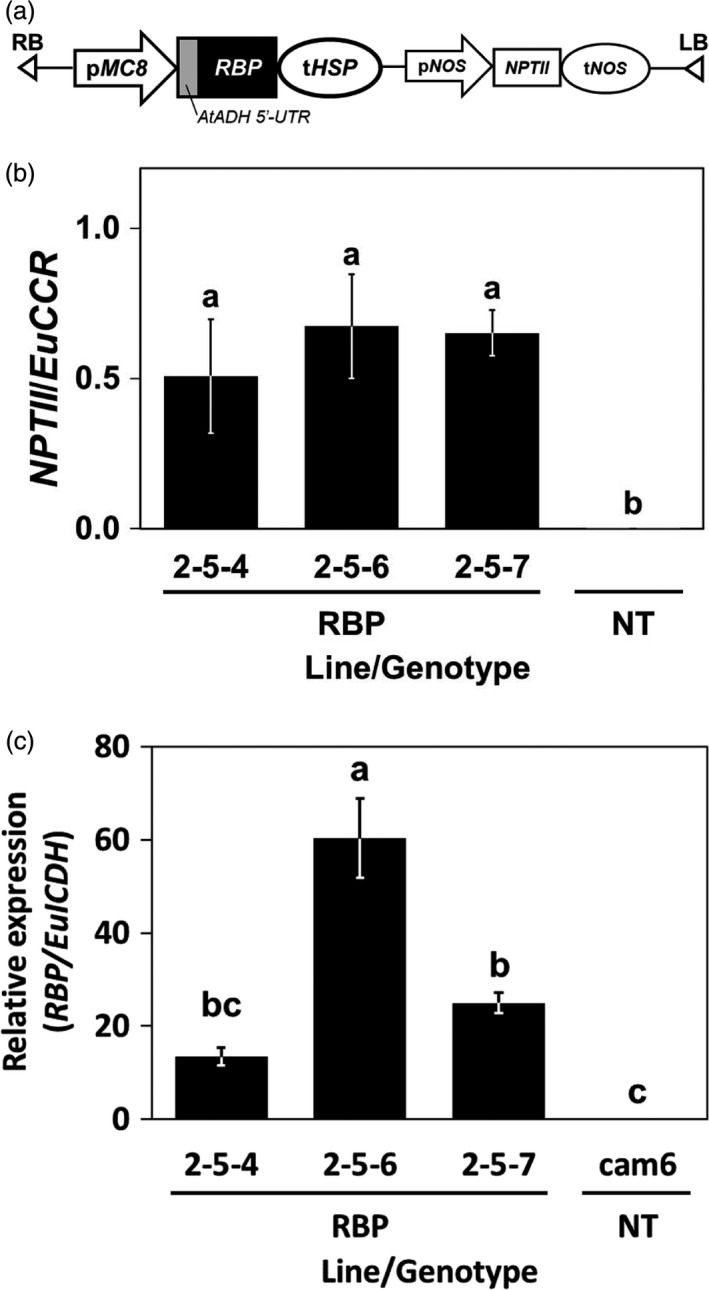
The structure, genomic integration, and expression of the transgenes. (a) Schematic diagram of the T‐DNA region. *AtADH* 5′‐UTR,* AtADH* transcriptional enhancer; *NPTII*,* neomycin transferase II*; pMC8, MC8 promoter; p*NOS* and t*NOS* ,* nopaline synthase* promoter and terminator; RB and LB, right and left border; t*HSP* ,* heat‐shock protein* terminator. (b) The estimated copy number of genomic integration of T‐DNA by qgPCR. (c) The expression level of *McRBP* estimated by qRT‐PCR. RBP and NT in panels (b and c): Transgenic plants harbouring *McRBP* and non‐transgenic plants, respectively. Error bars in (b and c) Standard error of three biological replications. The different letters on the top of the bars indicate significant differences among clones by the Tukey‐HSD test (α = 0.05).

The transgenic *E. camaldulensis* plants were transformed with the T‐DNA construct shown in Figure 2a by the Agrobacterium‐mediated transformation method (NEDO, 2013), and we chose three clones of them for the following experiment. To evaluate the stability of the genomic integration and the expression of the transgene, we performed a quantitative genomic PCR (qgPCR) and quantitative reverse transcription PCR (qRT‐PCR), respectively (Figure 2b,c).

In the qgPCR analysis, three plants randomly selected for each of the transgenic *E. camaldulensis* clones were tested. Amplifications of *NPTII* included in the T‐DNA were detected from all of the transgenic plants examined. As the endogenous reference gene for the calibration, we chose *Cinnamoyl‐CoA reductase* (*CCR*) gene, which is a single copy and shows low diversity in the genus *Eucalyptus* (McKinnon *et al*., 2008). The calculated ratios of the quantitation of *NPTII* to the quantitation of an endogenous single copy gene are shown in Figure 2b. The results indicated that all three transgenic clones had T‐DNA in their genome and were estimated to contain one haploid of T‐DNA in the respective genome (Figure 2b). Figure 2c shows the expression levels of *McRBP* in leaves of the transgenic *E. camaldulensis* tested by qRT‐PCR. The results demonstrated that all three transgenic clones expressed *McRBP* gene (Figure 2c). Our comparison of the three transgenic clones revealed that the expression levels of the 2‐5‐4 line and the 2‐5‐7 line were in nearly the same range and that the expression level of the 2‐5‐6 line was 2.4 times higher than theirs (Figure 2c). These results indicated that the transgenes were stably conserved in the three transgenic *Eucalyptus* clones, and a haploid of genomic integration was confirmed in each of the three transgenic clones.

### Assessment of severe salinity stress tolerance

**Figure 3:**
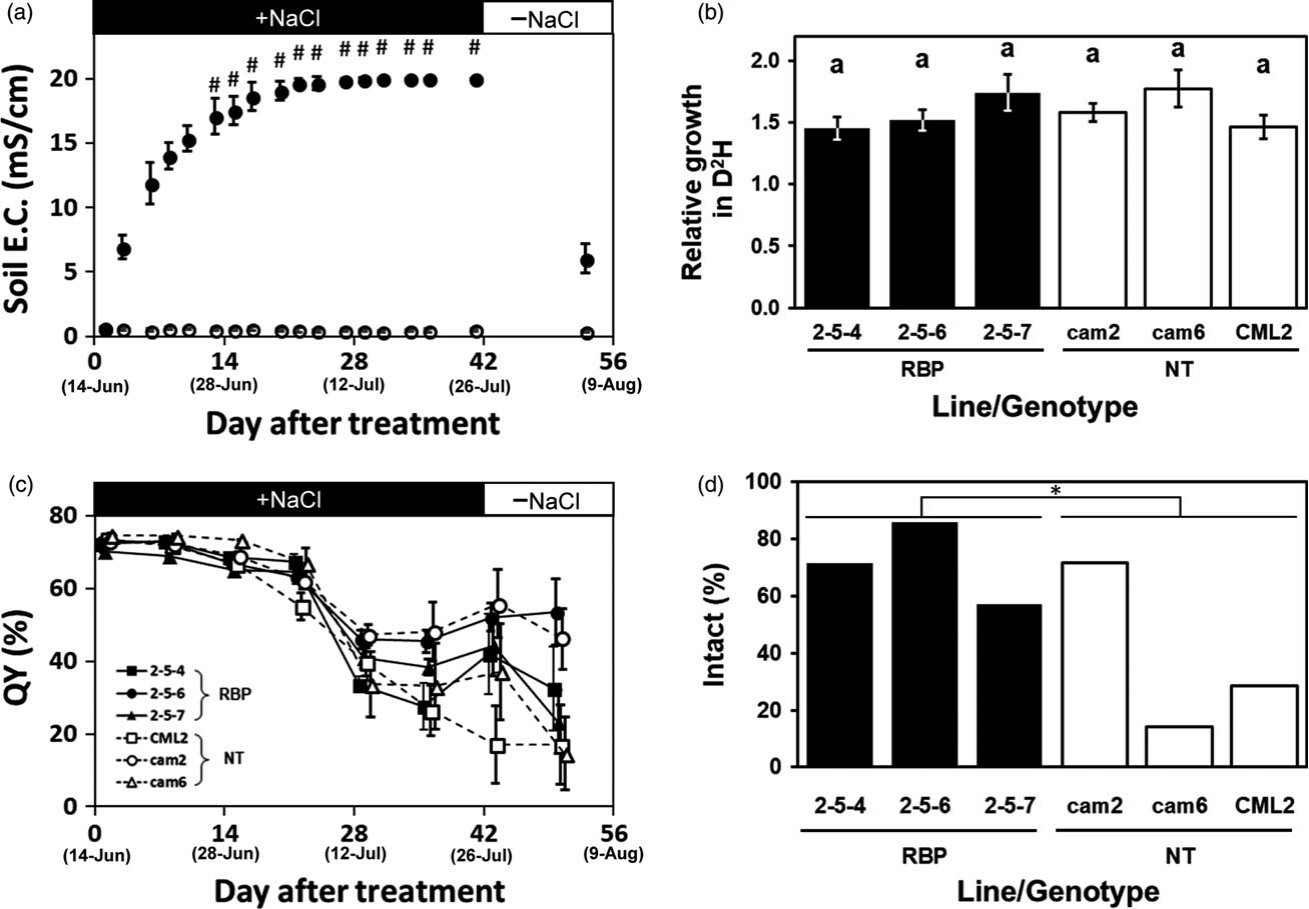
Salt tolerance evaluation following the high‐stringency salt stress treatment. (a) Changes in the electrical conductivity (EC) of soil treated with 400 mM NaCl. Filled and open plots: The mean EC for the treatment and control, respectively. #: The mean was calculated from measurements including saturation(s). (b) Relative growth in D^2^H after the 6‐week severe salinity stress treatment. (c) The changes in the quantum yield of photosynthesis (QY). Filled and open plots: The mean QY of transgenic and non‐transgenic plants, respectively. (d) The ratios of plants with intact apical buds after the 6‐week treatment. Filled and open bars: The ratio of transgenic (RBP) and non‐transgenic plants, respectively. *P =* *0.0209 between the transgenic and non‐transgenic groups by χ^2^ test. Error bars in (a, b) standard error.

With the objective of determining the plants’ acute tolerance to a severe‐stringency salt stress condition, we cultivated transgenic and non‐transgenic *Eucalyptus* on soil treated with 400 mM NaCl. In the first week of 400 mM NaCl treatment, the soil EC in the pot increased quickly to an average of approx. 14 mS/cm (Figure 3a). By the second week of the treatment, the local EC value exceeded 20 mS/cm (which is the measurable limit of the device), and the EC value at almost all measurement points exceeded 20 mS/cm after 4 weeks (Figure 3a). After 6 weeks of the treatment, the soil EC of the culture pots sharply decreased to approx. 6 mS/cm within 2 weeks by re‐watering with tap water (Figure 3a). Because the soil EC value reached up to 14 mS/cm within 1 week, all plants regardless transgenic or non‐transgenic plants stopped the growth (Figure 3b).

We used the quantum yield of photosynthesis (QY) as an indicator of plant health. The means of the leaf QY values slowly declined from 70.1%–74.6% to 55.1%–67.5% in the first 3 weeks, then plummeted to 33.6%–47.3% at the fourth week (Figure 3c). The means of the leaf QY values at the final (6th) week were 17.1%–55.8%, and there was no significant difference among the clones regardless of their transgenic or non‐transgenic status (Tukey‐HSD test, α=0.05) (Figure 3c). In addition, the leaf QY value did not recover within a short period by re‐watering with tap water (Figure 3c).

The ratios of plants with intact apical buds after the 6‐week 400 mM salt treatment are shown in Figure 3d. The ratios of the non‐transgenic plants were 14.2%–71.4%, and the mean was 38.1% (Figure 3d). In contrast, the ratios of the transgenic plants were 57.1%–85.7% and the mean was 71.4%, which was significantly higher than that of the non‐transgenic plants (χ^2^ test; *P* = 0.0209; Figure 3d). The appearance of the plants after the 6‐week treatment and 10‐day re‐watering with tap water showed the difference more clearly. Photos of typical plants are provided in Figure 5a. While almost all of the leaves of the non‐transgenic plants withered, the leaves of the transgenic plants showed some curling and partial withering but had remained mostly green (Figure 5a). These results indicated that *McRBP* gene improved the *E. camaldulensis* plants’ acute tolerance against severe‐stringency salinity.

### Assessment of moderate salinity stress tolerance

**Figure 4:**
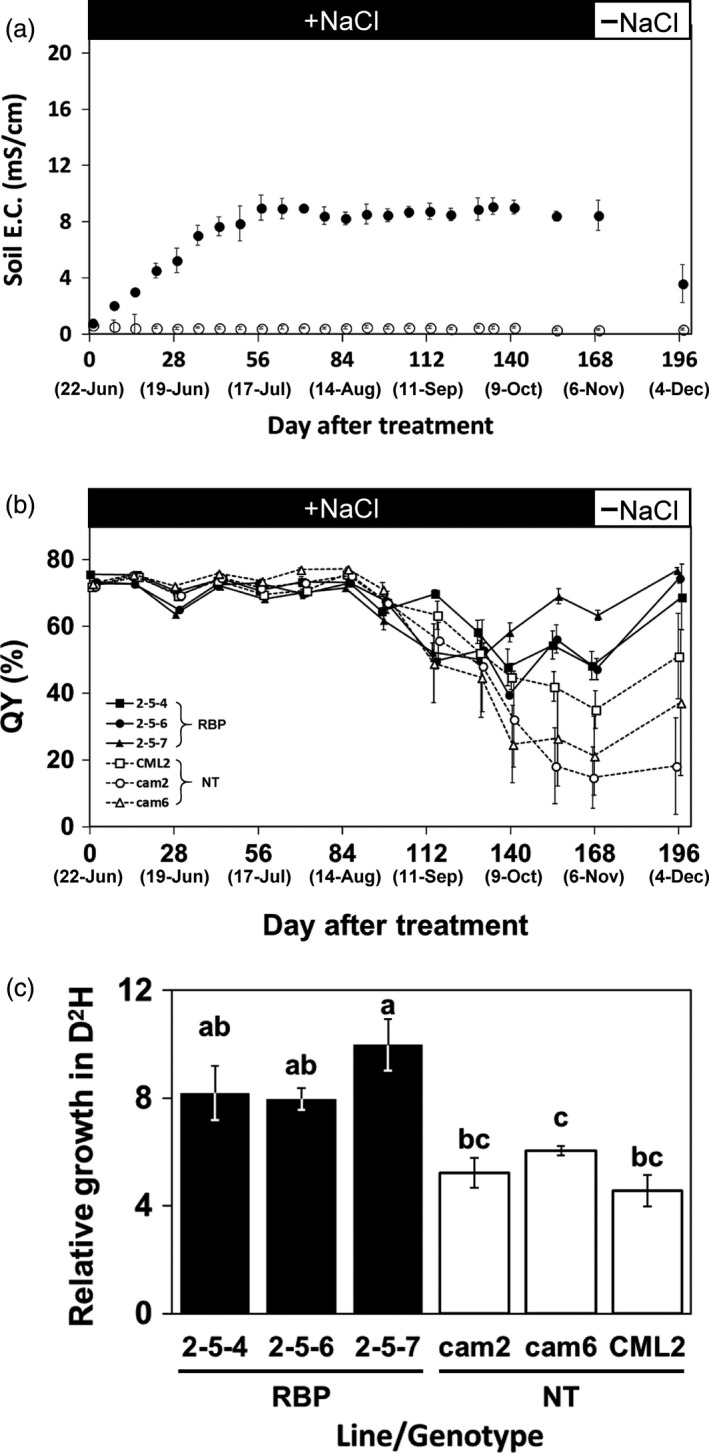
Salt tolerance evaluation by the moderate‐stringency salt stress treatment. (a) Changes in the electrical conductivity (EC) of soil treated with 70 mM NaCl. Filled and open plots: The mean EC for the treatment and control, respectively. (b) Changes in the quantum yield of photosynthesis (QY). Filled and open plots: The mean QY of the transgenic and non‐transgenic plants, respectively. (c) Relative growth of D^2^H after the 24‐week moderate salinity stress treatment. Filled and open bars: The ratio of transgenic (RBP) and non‐transgenic plants, respectively. The different letters on top of the bars: Significant differences among clones by the Tukey‐HSD test (α = 0.05). Error bars in (a, b) standard error.

With the objective of assessing salt tolerance under simulated conditions reflecting salinity land that would be considered as future plantation and/or afforestation sites, we conducted another cultivation trial of transgenic and non‐transgenic *Eucalyptus* on soil treated with 70 mM NaCl, i.e., moderate salinity. In the first 8 weeks of the treatment, the soil EC in the pots slowly increased to an average of 9.0 mS/cm and fluctuated slightly in the range of 8.2–9.1 mS/cm over the next 16 weeks until the treatment was terminated (Figure 4a). We confirmed that the soil EC of the culture pots decreased to approx. 3.5 mS/cm within 1 month after the end of the treatment by re‐watering with tap water (Figure 4a).

The mean leaf QY values of all plants regardless of transgenic and non‐transgenic status fluctuated slightly in the range of 63.7%–77.3% for the first 2 months and tended to decline over the subsequent 8 weeks (Figure 4b). After 20 weeks, the leaf QY values of the transgenic plants tended to have recovered slightly, whereas that of the non‐transgenic plants remained at low levels without recovery. (Figure 4b). At the end of the treatment, the mean leaf QY values in the transgenic and non‐transgenic plants were in the ranges 47.3%–63.3% and 14.8%–35.2%, respectively (Figure 4b), which is significantly different by the one‐way ANOVA (α = 0.001).

**Figure 5:**
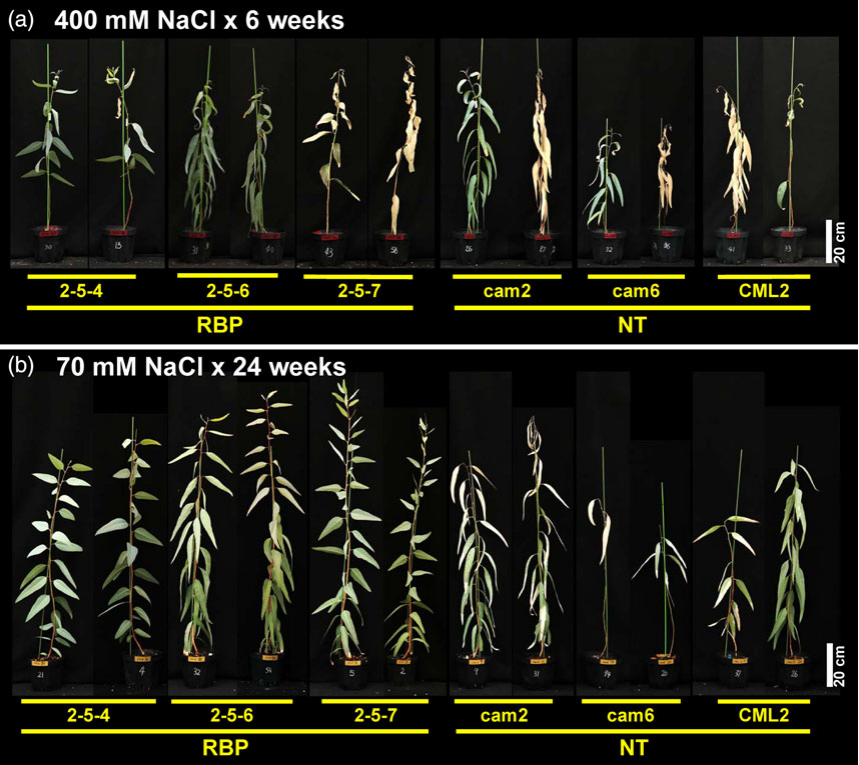
Photos of typical plants after salinity stress treatment. (a) Typical plants after 6‐week severe salt stress assay. (b) Typical plants after 24‐week moderate salt stress assay. 2‐5‐4, 2‐5‐6, and 2‐5‐7 are transgenic plants harboring *McRBP*; cam2, cam6, and CML2 are non‐transgenic plants. Scale bar: 20 cm.

During the approx. 6 months of treatment, all plants regardless of transgenic and non‐transgenic status survived. The appearance of the plants post‐treatment is shown in Figure 5b. We observed clear damage considered to be caused by the salt stress in non‐transgenic plants but not in transgenic plants (Figure 5b).

Figure 4c shows the biomass productions of the transgenic and non‐transgenic plants after the salt stress treatment. The relative biomass production in D^2^H of the transgenic plants in the treatment was 8.0–10.0 and was 1.6 times higher on average than that of the non‐transgenic plants at 4.6–6.0 (Figure 4c). The relative biomass production of the control‐group transgenic and non‐transgenic plants treated with tap water was in the range of 12.7–19.5, and no significant difference between these two groups in relative biomass production was shown by the one‐way ANOVA (α = 0.05).

**Figure 6:**
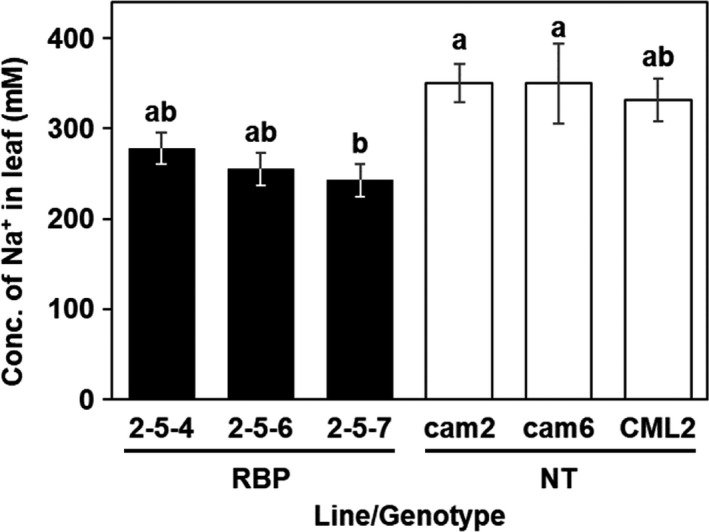
Sodium ion accumulation in the leaves of plants after 15 weeks of moderate‐salinity stress treatment. Filled and open bars: The mean concentration of sodium ion in transgenic (RBP) and non‐transgenic plants, respectively. Different letters on top of the bars: significant differences among clones by the Tukey‐HSD test (α = 0.05).

We also evaluated the accumulation levels of sodium ions in the plants’ leaves after 15 weeks of the salt treatment (Figure 6). The mean sodium ion concentration in the leaves of the transgenic plants was 242.8–278.3 mM, which is 25% lower on average than that of the non‐transgenic plants at 331.8–350.5 mM (Figure 6). These results suggested that in the transgenic *E. camaldulensis*,* McRBP* expression helped maintain the health of the plants and suppressed the loss of biomass production under the moderate salinity stress condition.

## Discussion

### McRBP function and abiotic stress tolerance

In this study, the *McRBP* gene was used as the trait gene for improving *E. camaldulensis*. *McRBP* was isolated from the common ice plant, *M. crystallinum*, as a candidate for a salt‐tolerant gene (Yamada *et al*., 2011), and encoded a protein having two RNA recognition motifs designated RRM_1 (PF00076) (Figures 1 and S1). The proteins containing RRM_1 motifs form a huge group, and there are more than 1 30 000 protein sequences from more than 1900 species ranging from bacteria to mammalians and higher plants in the Pfam database. Especially among higher plants, the diversity is very large; more than 300 and 550 RRM proteins were registered in the Pfam database for *A. thaliana* and rice, respectively. At the same time, the architecture and structure of the RRM proteins are also very diversified, i.e., over 1700 architectures and more than 800 structures were registered in the Pfam database, and it is difficult to predict their function based only on the conservation of the RRM_1 motif. Therefore, we performed our conservation analysis based on the conservation in motif structural units (Mihara *et al*., 2010) and searched for proteins with the most similar structure to McRBP from *A. thaliana* RRM proteins. The conservation analysis identified AtCP31A and AtCP31B as the proteins most similar to McRBP. AtCP31A and AtCP31B contained two RRM_1 motifs in the C‐terminal regions (Figure S1). In addition, we identified their homologous proteins in green alga (*Ostreococcus tauri*), lycophyte (*Selaginella moellendorffii*), eudicots (*Vitis vinifera*,* E. camaldulensis*,* E. grandis*), and monocots (*Oryza sativa*,* Sorghum bicolor*) and calculated/drew the molecular dendrogram (Figure S1a). The dendrogram revealed that there were each two copies each of the CP31 homologs in *Arabidopsis*, grape, *Eucalyptus*, rice, and *Sorghum*, but no CP31 homolog in green alga or *Selaginella* (Figure S1). These results indicated that the CP31 protein would be evolutionally conserved in higher plants and have conserved functions. Although only one CP31 homolog has been found in the ice plant, another CP31 homolog has been predicted to exist in other higher plants. From another point of view, the existence of an endogenous CP31 homolog in *Eucalyptus* species founded by the molecular analysis suggested the possibility of a future selection breeding program focused on the endogenous CP31 homolog.

A previous study suggested that AtCP31 binds to multiple chloroplast RNAs under a low temperature stress environment and plays a role in abiotic stress tolerance (Kupsch *et al*., 2012). It was also suggested that the RRM_1 motif preserved in CP31 binds non‐specifically to RNA (Kupsch *et al*., 2012; Tillich *et al*., 2009). It is known that non‐specific RNA‐binding proteins involved in such stress tolerance contribute to the maintenance of RNA stability under stress conditions due to RNA chaperone activity (Castiglioni *et al*., 2008; Jiang *et al*., 1997; Rajkowitsch *et al*., 2007; Rennella *et al*., 2017). In fact, the Monsanto Company reported that the expression of bacterial genes encoding an RNA chaperone protein enhanced the abiotic stress tolerance in *A. thaliana*, rice, and maize, and Monsanto has succeeded in commercializing a genetically modified maize event, MON87460, harbouring an RNA chaperone gene, *CspB*, derived from *Bacillus subtilis*, as a drought‐tolerant variety (Castiglioni *et al*., 2008). In the present study, the transcription anti‐termination assay suggested that McRBP exhibits RNA chaperone activity dependent on the conserved RRM_1 motif (Figure 1). Therefore, an *McRBP* gene that was isolated by functional screening for salt‐tolerance genes and encoded a protein having novel RNA chaperone activity was transformed into *Eucalyptus* in an attempt to improve abiotic stress tolerance, especially salinity tolerance.

In present study, we observed that elevations of Na^+^ in leaves were smaller in *McRBP* transgenic than that in non‐transgenic trees after the 6‐month moderate salt stress treatment (Figure 6). Maintaining intercellular ion homeostasis in a stress environment also includes transcriptional regulation of stress responsive genes including ion importer channels, exporter channels, transporters, and so on (Deinlein *et al*., 2014; Hanin *et al*., 2016; Hasegawa, 2013; Zhu, 2003). We speculated that McRBP might contribute to the maintenance of cellular ion homeostasis under the stress conditions by increasing the stability of transcriptional regulations of stress responsive gene expression and conferred to improve the growth in stress condition. Future experiments like as the transcriptional analysis would provide more information about the relationship between *McRBP* expression and the transcriptional stability in plant cell in the stress condition.

### *McRBP* expression improved *Eucalyptus*’ salt tolerance in both severe and moderate salt stress treatments

In this study, we used a novel salt tolerance test method in the pot cultivation. As the method for evaluating salt tolerance in a greenhouse, assessments of tolerance by determining the survival rate after short‐term high‐concentration saltwater treatment have been common, and we have evaluated some transgenic *Eucalyptus* by this method (Yu *et al*., 2009, 2013d). This method is advantageous in that the time required for the evaluation is short and the influence of other environmental factors is small. On the other hand, it was suggested that the survival in severe stress conditions and the growth performance in moderate stress conditions often encountered in the environment are not equal (Skirycz *et al*., 2011). The method we have described herein enabled the maintenance of the soil EC value within a targeted range depending on the concentration of saltwater used for the treatment; e.g., the range of around 8–9 with 70 mM NaCl treatment (Figure 4a). According to a classification of salt‐damaged soil, soil treated with 70 mM NaCl mimics the saline soil (Grigore and Toma, 2017; Richards, 1954) that comprises approx. 60% of the total global salt‐affected soil, at >650 000 hectares (Wicke *et al*., 2011). Our present findings indicate that the transgenic *E. camaldulensis* harbouring *McRBP* can grow in a saline soil environment, and that these trees may be a candidate for future practical applications in forestry plantation and/or environmental afforestation.

From the predicted McRBP function, it was assumed that there would be no morphological change by insertion of the *McRBP* gene in *Eucalyptus*. In fact, there was no remarkable difference in leaf morphologies among the transgenic and non‐transgenic lines (Figure S2). On the other hand, the leaf angles of the transgenic lines 2‐5‐4 and 2‐5‐7 tended to be slightly different from those of the other transgenic and non‐transgenic clones (Figure S3a,b,e,f). The 2‐5‐6 trees did not exhibit these morphological changes (Figure S3a,b,e,f). However, trees with these characteristics were observed in some individuals of the non‐transgenic *E. camaldulensis* population. The genetic backgrounds of the transgenic *E. camaldulensis* generated by the method used in this study were heterozygotic, because hypocotyls of the seedlings derived from the bulk seeds were used as the explants in the transformation experiments. We also observed similar morphological characteristics in the transgenic *E. camaldulensis* harbouring another transgene but generated around the same time (Tran *et al*., 2018). From these facts, we considered that the morphological characteristics of 2‐5‐4 and 2‐5‐7 were not caused by *McRBP* expression but rather by polymorphism within the bulk seeds.

It was observed that the transgenic *E. camaldulensis* trees had superior tolerance under both severe and moderate stringency salinity stress conditions, i.e., treatment with 400 and 70 mM NaCl, respectively. Under both conditions, clear differences were apparent in the appearance of the plants after the treatments (Figure 5). Under the severe stress treatment, some plants showed withered apical buds at the fourth week, and the growth of all the transgenic and non‐transgenic plants was stopped by the treatment. However, the ratios of plants with intact apical buds at the final measurement time point differed significantly between the pooled transgenic plants and pooled non‐transgenic plants (Figure 3d). We previously performed the same severe stress treatment for the transgenic *E. camaldulensis* harbouring the *codA* gene and observed no significant difference between the transgenic and non‐transgenic groups. These results would suggest that *McRBP* is superior to *codA* as a transgene conferring acute severe stress‐resistance to *E. camaldulensis*, although other factors, such as the transcriptional levels and the insertion positions of their transgenes, should also be considered.

Under the moderate stress treatment, all the transgenic and non‐transgenic plants survived, and a significant difference in growth was observed between the transgenic and non‐transgenic plants (Figure 4c). It was reported that a transgenic *E. camaldulensis* harbouring a *codA* gene—which was derived from the soil bacterium *Arthrobacter globiformis* (Ikuta *et al*., 1977) and encodes an enzyme related to the synthesis of glycine betaine, an osmoprotectant (Ashraf and Foolad, 2007; Chen and Murata, 2008; Giri, 2011; Kurepin *et al*., 2015)—also survived and showed a significant reduction of growth decline due to salt stress compared with the non‐transgenic *E. camaldulensis* lines (Tran *et al*., 2018). Relative growth under 6‐month salt treatment was higher in the *McRBP* transgenic lines than the *codA* transgenic lines—i.e., the relative growths of the *McRBP*,* codA*, and non‐transgenic lines were in the ranges of 8.0–10.0, 6.8–7.7, and 4.6–6.0, respectively (Figure 4c; Tran *et al*., 2018). These results would suggest that *McRBP* is superior to *codA* as a transgene conferring chronic moderate stress‐tolerance to *E. camaldulensis*, but other factors, such as the transcriptional levels and the insertion positions of their transgenes, should also be considered. Our comparison of the results from the two levels of salt conditions suggested that evaluations based on growth under the moderate salt condition would be preferable for the development of abiotic stress‐tolerant plants for practical use.

### Consideration on environmental impacts of transgenic *Eucalyptus camaldulensis* harbouring *McRBP*

The first approval was given to *Eucalyptus* for environmental release for commercial use in Brazil, which has one of the largest areas of *Eucalyptus* plantation forest (Ledford, 2014). This approval was expected to accelerate the future applications of biotech forestry plantation trees (Häggman *et al*., 2013). The transgenic *E. camaldulensis* trees used in the present study constitutively expressed two transgenes, *McRBP* and *NPTII*. *NTPII* has already undergone a sound‐science risk assessment, and it was internationally agreed that the expression of *NPTII* causes no considerable risk to human health of the environment (EFSA, 2007; OGTR, 2017). Regarding McRBP, it is expected that McRBP has a role in the enhancement of the abiotic stress tolerance via RNA chaperon‐like activity, but McRBP has no enzyme activity. We confirmed that McRBP does not contain a suspected allergenic sequence by conducting a database search (Allergen Database for Food Safety, provided by the National Institute of Health Science, Japan; (Nakamura *et al*., 2014, 2005). Based on this information, we hypothesize that McRBP presents no appreciable environmental risk to biodiversity. Environmental risk assessments of the transgenic *E. camaldulensis* trees in field trials are necessary prior to their environmental release.

## Materials and methods

### Transcription anti‐termination *in vivo* assay

The RNA chaperone activity of the McRBP protein was confirmed by a transcription anti‐termination assay, in which the reporter gene was replaced from the *chloramphenicol acetyltransferase* (*cat*) gene to the *neomycin phosphotransferase* (*NPTII*) gene (Phadtare *et al*., 2003) (Figure 1a). As the reporter plasmid, the strong ρ‐independent *trpL* terminator inserted upstream of the *NPTII* gene usually folds into a hairpin structure and terminates the transcription, and the RNA chaperone effector unfolds the hairpin structure and transcribes the downstream *NPTII* gene (Phadtare *et al*., 2003) (Figure 1a). The reporter plasmid without a *trpL* terminator was used for the control (Figure 1a). As the effector plasmid, a plasmid containing an expression cassette consisting of the full length of *McRBP* (RBP) was used. The same plasmid but containing an expression cassette of *McRBP* with mutations in the conserved amino acids (mRBP) (Figure 1b) or the *GFP* gene was used as a negative control. The reporter plasmids and/or the effector plasmids were transformed into *E. coli* strain BL21(DE3), and then were pre‐cultured in liquid 2YT medium containing 1 mM isopropyl β‐d‐1‐thiogalactopyranoside (IPTG) at 37 °C with shaking at 35 rpm by a shaking incubator with auto‐turbidity and a bio‐photorecorder (Advantec Toyo Kaisha, Tokyo, Japan) until the cell density reached the range of 2.0–3.0 (A_600_). Then, the bacterial cultures were diluted and spread on a 2YT‐agar plate containing 1 mM IPTG and 20 mg/L kanamycin. After incubation at 37 °C overnight, the colonies were counted, and the relative colony formation units were calculated.

### Plant materials and cultivation condition

The transgenic *E. camaldulensis* were generated by the Agrobacterium‐mediated method with a Ti‐plasmid vector (Figure 2a), and we introduced the expression cassette of *McRBP* and *NPTII* (NEDO, 2013). We chose three clones of transgenic *E. camaldulensis* harbouring *McRBP* (2‐5‐4, 2‐5‐6, and 2‐5‐7) for the semi‐confined screen‐house trials. Because these transgenic lines were regenerated from seedlings derived from a bulk of seed that was genetically heterogeneous, their genetic backgrounds were diverse. Thus, the near‐isogenic lines of the transgenic plants were not available. From these situation, three independent non‐transgenic lines of *E. camaldulensis* (cam2, cam6, and CML2) were used as the controls (Yu *et al*., 2009). Propagation of plantlets used for the screen house trials were performed by the stem cutting method described in our previous report (Tran *et al*., 2018). The plantlets were pre‐cultivated in screen house for approximated 9 months and used for the trials from May 2017. The detail condition on the screen house cultivation was described in the previous reports (Tran *et al*., 2018).

### Quantitative genomic polymerase chain reaction

The young leaves were collected, and we extracted genomic DNA from them by using a DNeasy^®^ Plant Mini Kit (Qiagen, Hilden, Germany). For the qgPCR, the reaction volume of 10 μL contained 5 μL of Thunderbird™ SYBR^®^ qPCR Mix (Toyobo, Osaka, Japan), 0.3 μmol/L each of the primer pair, 1X ROX, and an aliquot of the template. The qgPCR reaction was performed with an ABI PRISM 7900 (Thermo Fisher Scientific, Waltham, MA) according to the following step‐cycle program: pre‐incubation at 95 °C for 10 min, follow by 40 cycles of denaturing at 95 °C for 15 s, and annealing and extension at 60 °C for 1 min each cycle. The primer pairs for the qgPCR of the copy number estimation were as follows: 5′‐TGAATGAACTGCAGGACGAG‐3′ and 5′‐TTCAGTGACAACGTCGAGCA‐3′ for *NPTII* as the transgene, and 5′‐CTCGAACGAATGGAGTCGCT‐3′ and 5′‐TGAGAACGGACCCAGTCGTA‐3′ for *CCR* as the host plant endogenous gene (McKinnon *et al*., 2008). The plasmid subclones of both *NPTII* and *CCR* amplicon fragments were used as the calibrator of the quantitation. The ratio of the genomic integration of the transgene was estimated based on the ratio of the quantity of transgene to that of the endogenous gene.

### Quantitative reverse transcription PCR

Transcription levels of transgene were analyzed by qRT‐PCR described in our previous report (Tran *et al*., 2018) i.e. total RNA was extracted from young leaves with an RNeasy^®^ Plant Mini Kit (Qiagen), reverse transcription (RT) reactions were performed using ReverTra Ace^®^ qPCR RT Master Mix with gDNA Remover (Toyobo), and qRT‐PCR were performed Thunderbird™ SYBR^®^ qPCR Mix (Toyobo) reagent and an ABI PRISM 7900 (Thermo Fisher Scientific) instruments. The primer pairs for qRT‐PCR were as follows: 5′‐TTGCTGCTCTTGATGGACAG‐3′ and 5′‐AAACGCACGTCTTGGTCTTT‐3′ for *McRBP*, and 5′‐TTGCTGCTCTTGATGGACAG‐3′ and 5′‐AAACGCACGTCTTGGTCTTT‐3′ for *Eucalyptus isocitrate dehydrogenase* gene (*ICDH*) as the endogenous reference gene for expression analysis (Boava *et al*., 2010; Oguchi *et al*., 2014). The plasmid subcloned of both *McRBP* and *ICDH* amplicon fragments were used as the calibrator of the quantitation. The expression of *McRBP* transgene was calculated based on the ratio of the quantity of transgene to that of *ICDH* gene.

### Salt tolerance assay in severe stress treatment

Seven of individual plants per clonal line were provided for the assay of severe salt stress treatment. The young trees used for the experiments were transferred to slit pots, which allow water to rise from the slits at the bottom of the pot to the soil surface within a short time (Tran *et al*., 2018). Soil pots were treated by salt water as the same manner described in our previous report (Tran *et al*., 2018), i.e., the pots were placed on a plastic container filled with water containing 400 mM NaCl until the water level rose from the bottom slits to the surface of the pot soil, three times per week. Trees in pots treated in the same manner but with the use of plain tap water were used as controls. The level of accumulated salt in soil was monitored using a FieldScout^®^ Direct Soil EC Probe (Spectrum Technologies, Aurora, IL) by the procedure described in our previous report (Tran *et al*., 2018). During this evaluation, the appearance of the plants was observed weekly, and we calculated the ratio of plants with no wilted leaf. We also measured the quantum yield of photosynthesis (QY) of the leaves every week, using a FluorPen FP100 instrument (Photon Systems Instruments, Drasov, Czech Republic) (Tran *et al*., 2018). The plant heights and basal diameters were measured at the starting and termination of this experiment, and we calculated the D^2^H index by the following formula to estimate the volume of woody biomass (Tran *et al*., 2018).

$$D^{2}H={\left(\text{Basal diameter}\right)}^{2}\times \left(\text{Plant height}\right)$$

### Salt tolerance assay in moderate stress treatment

Five individual plants per treatment and clonal lines underwent the assay following the salt stress treatment described in our previous report (Tran *et al*., 2018) as the moderate stress treatment; i.e. the slit pots with testing young trees were soaked in a plastic container filled with water containing 70 mM NaCl, three times per week. Trees in pots treated in the same manner but with the use of plain tap water were used as controls (Tran *et al*., 2018). In this assay, the plant heights and basal diameters were measured monthly, and we calculated the D^2^H index by the above formula.

The QY of the leaves was also monitored every 2 weeks in the same manner as that used for the severe salt stress treatment. After the 15‐week salt treatment, we evaluated the accumulation levels of sodium ions in the leaves. A healthy mature leaf (i.e., with a QY >70%) was collected from each of the plants, and we excised eight leaf disks from each leaf with the use of a cork borer (No. 2, φ = 5.5 mm). Four of the eight leaf disks were boiled in a 2‐mL tube with 1 mL of distilled water and then shaken vigorously. The sodium ion concentration of 200 μL of the clear supernatants was measured by the determination of the Na^+^ concentration by a Compact Na^+^ meter (LAQUAtwin B‐772; Horiba, Kyoto, Japan). The fresh and dried weights of the other four disks were identified. We then calculated the accumulation levels of sodium ions in each leaf by the following formula.

$${\text{Na}}^{+}\;\text{in leaf [m}M]=\frac{\left({\text{Na}}^{+}\;\text{conc. of sup.}\;\left[\mu \text{g/mL}\right]\right)\;÷\;\left(\text{MW of}\;{\text{Na}}^{+}\right)}{\left(\text{gFW of leaf disks})-(\text{gDW of leaf disks}\right)}\times 10^{3}$$

### Statistical analysis

The data were subjected to a statistical analysis using either a two‐way analysis of variance (ANOVA) or the split plot analysis of variance (Perry *et al*., 2009). Each ANOVA was performed using R ver. 3.4.0 software (2017‐04‐21) and/or Microsoft Excel 2016 MSO (16.0.4266.1001) (Microsoft, Redmond, WA). The Tukey‐Kramer multiple comparisons test was used as necessary, with R.

## Conflict of interest

The authors declare no conflict of interest.

## Supporting information


**Figure S1** McRBP‐related proteins in various plant species.
**Figure S2** Leaf morphology.
**Figure S3** Photos of typical plants before and after salinity stress treatment.
