# Supplementary material for: Development and evaluation of novel salt‐tolerant Eucalyptus trees by molecular breeding using an RNA‐Binding‐Protein gene derived from common ice plant (Mesembryanthemum crystallinum L.)
Source: Plant Biotechnol J. 2018 Oct 12;17(4):801–11. doi: 10.1111/pbi.13016 (PMC6419579; doi:10.1111/pbi.13016)
Supplement: Supplementary file 1 — Figure S1 McRBP‐related proteins in various plant species. Figure S2 Leaf morphology. Figure S3 Photos of typical plants before and after salinity stress treatment. [file PBI-17-801-s001.pdf]

Supplementary information

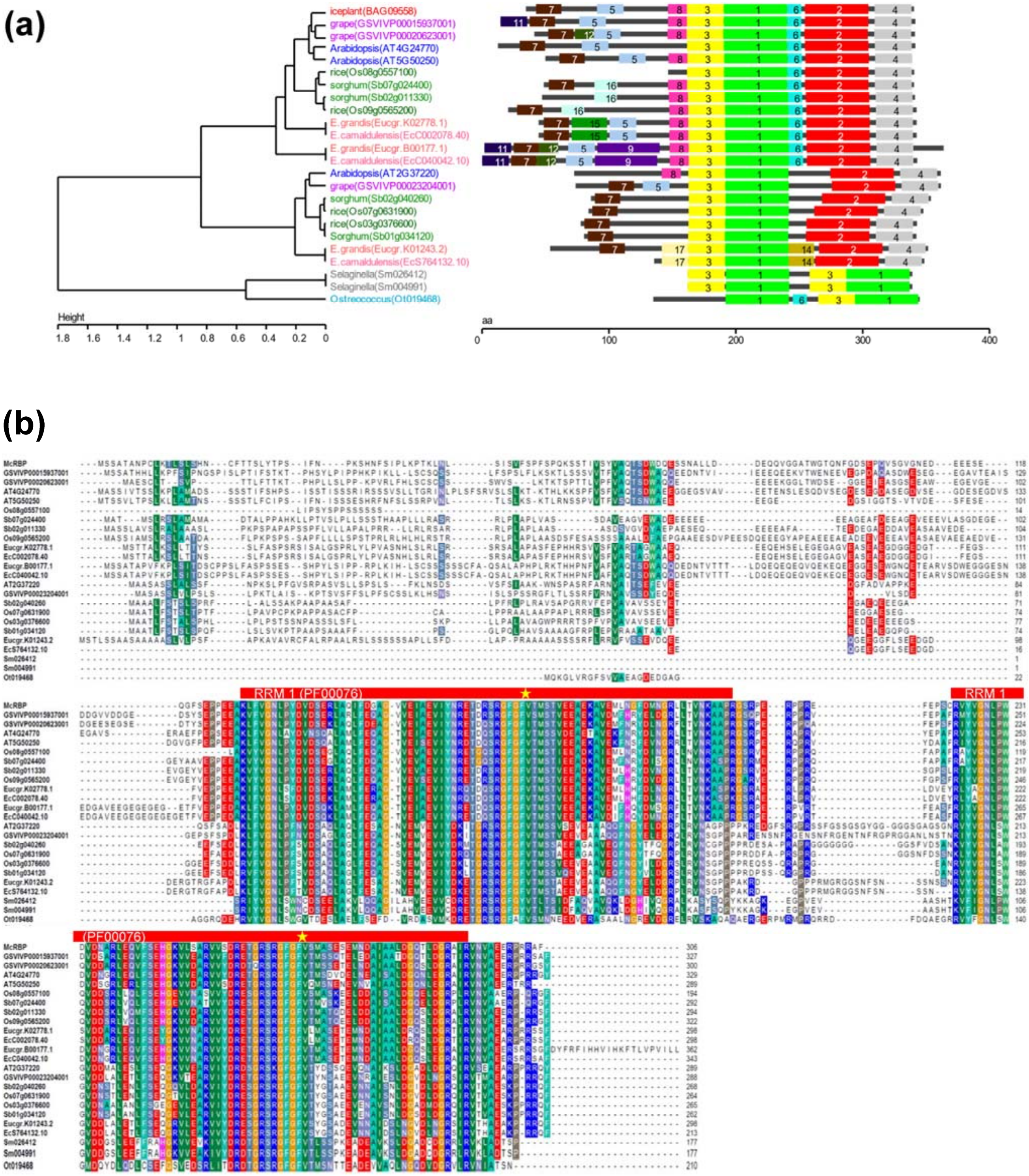

Suppl. Fig. S1 McRBP-related proteins in various plant species (a) Conserved motif-associating dendrogram of McRBP-related proteins, (b) Alignment of Amino acid sequence of McRBP-related proteins Prediction of candidates for orthologues of McRBP-related proteins were from *Arabidopsis thaliana*, rice (*Oryza sativa*), *Sorghum bicolor*, grape (*Vitis vinifera*), *Selaginella*

moellendorffii, *Ostreococcus tauri*.predicted by the surveyed conserved motif alignment diagram and the associating dendrogram (SALAD ver.3) (Mihara *et al.*, 2010). In addition, the candidates for orthologues of McRBP of *Eucalyptus camaldulensis* and *E. grandis* were found from Kazusa Genome Database and Phytozome by BLAST searches. The SALAD dendrogram were calculated and drew by the interactive SALAD analysis. The alignment was performed by ClustalW Multiple alignment. Red boxes above the alignments indicate consensus motif for RRM_1 (PF00076) and yellow stars indicate the mutation positions in the mRBP Effector in Figu.1.

(References) Mihara, M., Itoh, T., and Izawa, T. (2010). SALAD database: a motif-based database of protein annotations for plant comparative genomics. *Nucleic Acids Research,* **38**, D835-D842.

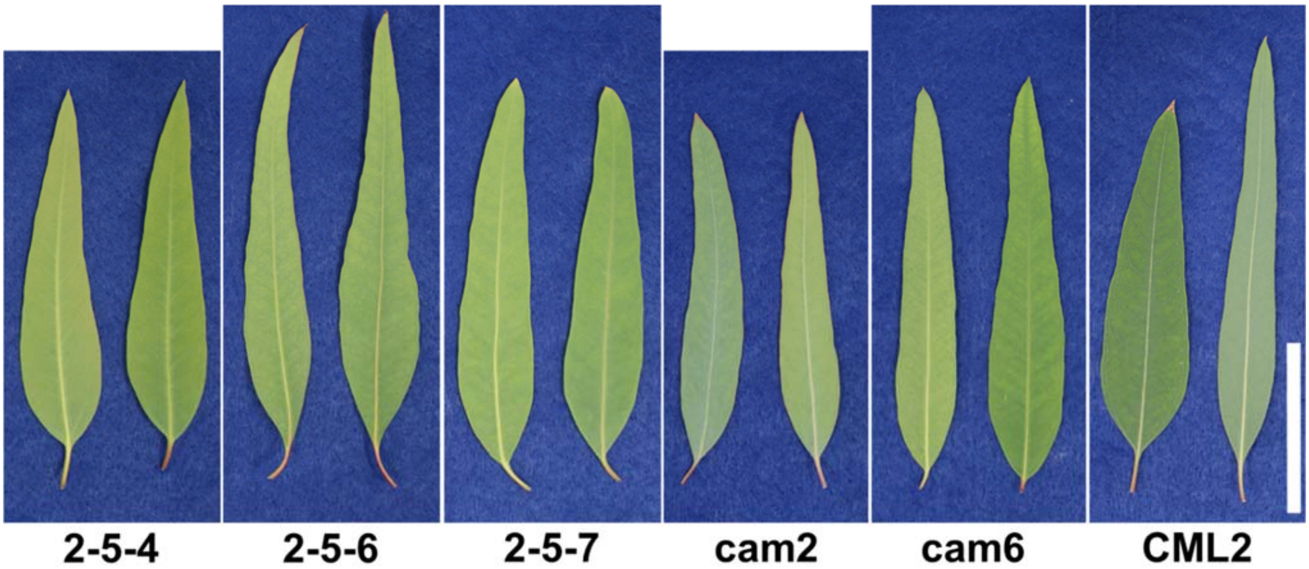

Suppl. Fig. S2 Leaf morphology Fully matured adult leaves were collected from RBP-transgenic and non-transgenic *E. camaldulensis* plants cultivated in the screen house for more than 1 year. Bar indicate 5 cm.

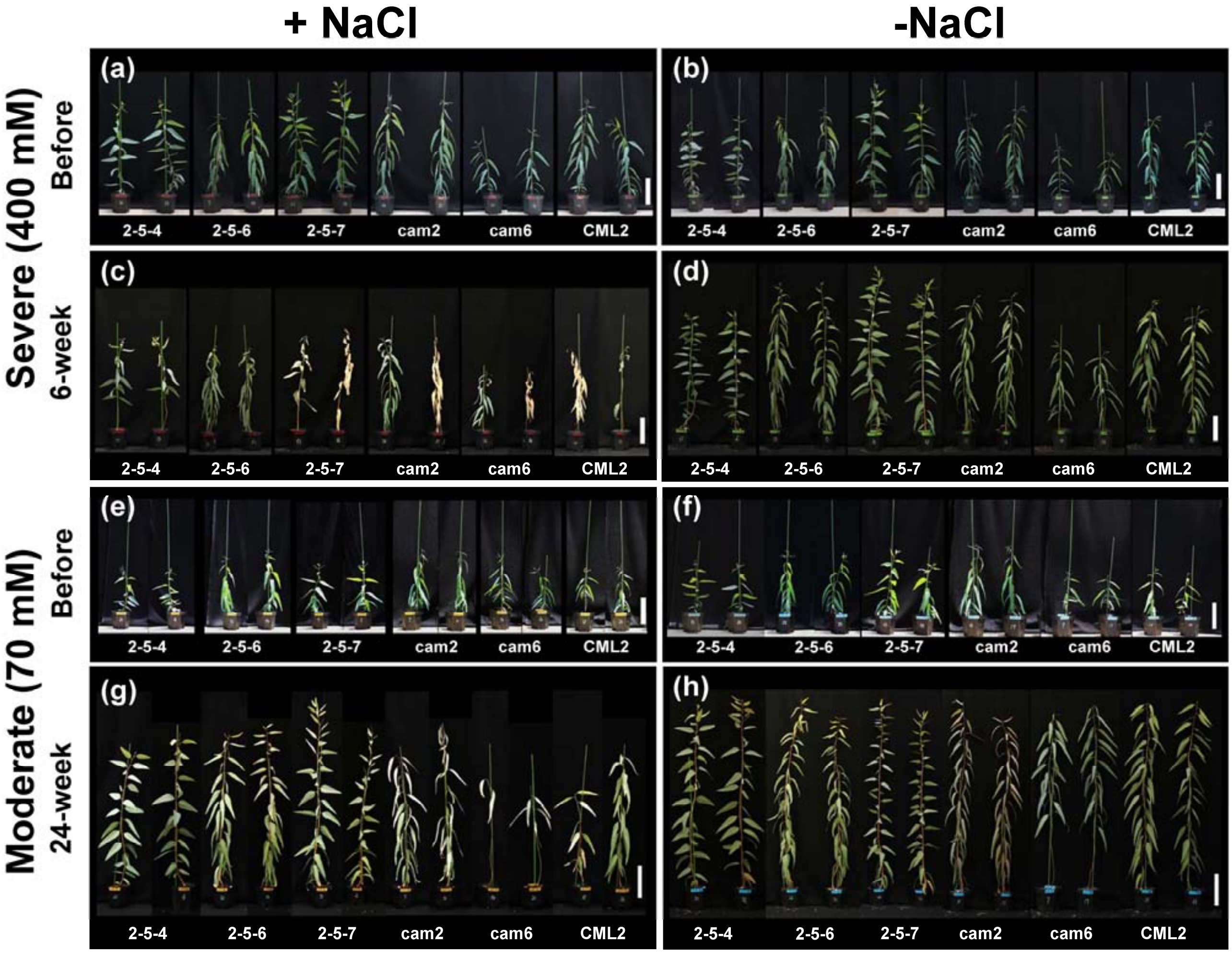

Suppl. Fig. S3 Photos of typical plants before and after salinity stress treatment. (a)-(d): Typical plants before and after 6-week severe salt stress assay. Plants before and after the six-week severe salt stress treatment for the respective transgenic and non-transgenic lines were shown in **(a)** and **(c)**, respectively. Plants before and after the six-week control treatment were shown in **(b)** and **(d)**, respectively. **(e)-(h):** Typical plants before and after 24-week moderate salt stress assay. Plants before and after the 24-week moderate salt stress treatment for the respective transgenic and non-transgenic lines were shown in **(e)** and **(g)**, respectively. Plants before and after the 24-week control treatment were shown in **(f)** and **(h)**, respectively. 2-5-4, 2-5-6 and 2-5-7 are transgenic plants harboring *McRBP*; cam2, cam6 and CML2 are non-transgenic plants. Scale bar: 20 cm.
